# Regulation of gap junctions by nitric oxide influences the generation of arrhythmias resulting from acute ischemia and reperfusion *in vivo*

**DOI:** 10.3389/fphar.2013.00076

**Published:** 2013-06-14

**Authors:** Ágnes Végh, Márton Gönczi, Gottfried Miskolczi, Mária Kovács

**Affiliations:** Department of Pharmacology and Pharmacotherapy, University of SzegedSzeged, Hungary

**Keywords:** ischemia/reperfusion, arrhythmias, gap junction, nitric oxide

## Abstract

Myocardial ischemia resulting from sudden occlusion of a coronary artery is one of the major causes in the appearance of severe, often life-threatening ventricular arrhythmias. Although the underlying mechanisms of these acute arrhythmias are many and varied, there is no doubt that uncoupling of gap junctions (GJs) play an important role especially in arrhythmias that are generated during phase Ib, and often terminate in sudden cardiac death. In the past decades considerable efforts have been made to explore mechanisms which regulate the function of GJs, and to find new approaches for protection against arrhythmias through the modulation of GJs. These investigations led to the development of GJ openers and inhibitors. The pharmacological modulation of GJs, however, resulted in conflicting results. It is still not clear whether opening or closing of GJs would be advantageous for the ischemic myocardium. Both maneuvers can result in protection, depending on the models, endpoints and the time of opening and closing of GJs. Furthermore, although there is substantial evidence that preconditioning decreases or delays the uncoupling of GJs, the precise mechanisms by which this attains have not yet been elucidated. In our own studies in anesthetized dogs preconditioning suppressed the ischemia and reperfusion-induced ventricular arrhythmias, and this protection was associated with the preservation of GJ function, manifested in less marked changes in electrical impedance, as well as in the maintenance of GJ permeability and phosphorylation of connexin43. Since we have substantial previous evidence that nitric oxide (NO) is an important trigger and mediator of the preconditioning-induced antiarrhythmic protection, we hypothesized that NO, among its several effects, may lead to this protection by influencing cardiac GJs. The hypotheses and theories relating to the pharmacological modulation of GJs will be discussed with particular attention to the role of NO.

## INTRODUCTION

Traveling on the London underground you may frequently hear “Mind the gap! Mind the gap! ” This warning call is also valid for the heart when the genesis of arrhythmias is considered. Gaps not only separate but also connect cells by forming special channels, termed gap junctions (GJs), which allow fast electrical and metabolic cross-talk between the neighboring cells. In myocardial tissue, these GJ channels are accumulated in clusters located in the intercalated disks, and they represent low resistance pathways between the adjacent cells, allowing fast spread of impulse from the one cell to the other (electrical coupling). These channels can also transfer small molecules (less than 1,000 Da) resulting in tight metabolic intercellular communication (metabolic coupling). Since, the shape of the ventricular cardiomyocytes is elongated and the GJs are preferably located in the longitudinal end of the cell, under normal conditions, the action potential is propagated in longitudinal direction (Spach et al., 1981; Rudy and Quan, 1987; Peters and Wit, 1998; Rohr, 2004). This uniform anisotropy that mainly results from the structural arrangement (longitudinal vs. transversal) and electrical properties of GJs (low resistance), makes possible that the heart behaves as an electrical syncytium. However, under pathologic conditions, such as the acute myocardial ischemia, as the consequence of the rapid metabolic changes (Shaw and Rudy, 1997), these GJs are uncoupled, resulting in the closure of the low resistance pathways and changes in impulse propagation. In homogeneity (non-uniform anisotropy) develops within the cardiac tissue as regards the electrical conduction, which leads ultimately to arrhythmia generation (Spear et al., 1992; De Groot and Coronel, 2004).

The present paper will focus on the role of GJs in the generation of ventricular arrhythmias due to acute myocardial ischemia. We will discuss how the pharmacological modulation of GJs would influence these ischemia-induced early ventricular arrhythmias, and put forth a hypothesis, based mainly on our own studies in anesthetized dogs, that nitric oxide (NO), an important endogenous modulator of heart function, may also regulate cardiac GJs. We provide evidence that the effect of NO on GJs might have a role in the cardioprotective (antiarrhythmic) effect of preconditioning and NO donors.

## THE ROLE OF GAP JUNCTIONS IN THE ACUTE ISCHEMIA-INDUCED VENTRICULAR ARRHYTHMIAS

There seems to be consensus in respect that arrhythmias occurring soon (within 3 min) after the onset of the coronary artery occlusion result from those ionic and electrophysiological changes which are due to the rapid switch of myocardial metabolism from aerobic to anaerobic mode (Janse et al., 1986). These metabolic changes (loss of ATP, fall in intracellular pH, accumulation of lactate, etc.) are apparent within seconds or minutes after the onset of ischemia and directly affect the function of ion channels and exchangers, resulting in considerable alterations in impulse generation and conduction (Cascio, 2001). Without going into details, conditions develop during this early phase of ischemia favor reentry, which is thought to be the main mechanism underlying the phase Ia arrhythmias (Kléber, 1983; Janse et al., 1986).

Although processes underlying generation of phase Ib arrhythmias are less well understood, there is no doubt that uncoupling of GJs play an important role. As is mentioned above in the uniformly anisotropic heart the transfer of an impulse is largely dependent upon the resistance of GJs, which is lower in longitudinal than transversal direction (Hoyt et al., 1989; Saffitz et al., 1995). This provides longitudinal preference over transversal conduction (Spach et al., 1981; Peters and Wit, 1998) and a safety for normal cell-to-cell impulse propagation (Spach and Heidlage, 1995). However, under ischemic conditions, particularly with the progression of ischemia, the further loss of ATP and intracellular K^+^, the accumulation of harmful metabolites and ions, the release of catecholamines, etc., would result in a milieu in which the uncoupling of GJs increases (White et al., 1990; Dhein, 1998). This leads to non-uniform changes in tissue resistance and inhomogeneous impulse conduction (Wojtczak, 1979; Kléber et al., 1987; Cascio et al., 2005) which initiate and maintain reentry during phase Ib (Spach et al., 1988). On the other hand, the increased resistance resulting from interruption of cell-to-cell coupling decreases the injury current, although at moderate levels of uncoupling this current would still be sufficient to induce delayed after-depolarization and trigger focal activity (Janse and van Capelle, 1982). Another consequence of the “metabolic overload” in the ischemic myocardium which largely accounts for the uncoupling of GJs is the reduced phosphorylation of connexin43 (Cx43), which is the primary structural protein of GJs in the ventricle (Söhl and Willecke, 2004). The ischemia-induced dephosphorylation of Cx43 results in conformational changes in connexin and leads to the closure of GJs and translocation of Cx43 from the membrane to the cytosol (Beardslee et al., 2000). This ischemia-induced Cx43 dephosphorylation and the subsequent closure of GJs occurs within 30 min (Beardslee et al., 2000; Schulz et al., 2003), making possible to use the measurement of Cx43 phosphorylation as a tool for the assessment of GJ function even during such a relatively short period of ischemia.

Functionally, GJ channels can be in open and closed state, although the conductance of a single channel may vary between several states – from closed, residual to the several levels of conducting (open)states – which are regulated by phosphorylation of the C-terminal of the connexin (Kwak and Jongsma, 1996). The assessment of GJ function particularly under *in vivo* conditions is rather difficult.Most of the currently used methods provide only indirect evidence on the coupling status of GJs. Measurement of GJ permeability using small molecular weight dyes (Ruiz-Meana et al., 2001) or the determination of connexin phosphorylation (Ando et al., 2005) allows evaluation of coupling only at a certain time point. Although measuring conduction velocity by activation mapping techniques (Rohr et al., 1998; Henriquez et al., 2001), or tissue impedance (resistivity and phase angle) changes by the use of a four-pin electrode method (Kléber et al., 1987; Cinca et al., 1997; Padilla et al., 2003) make possible continuous recording, these methods represent also only indirect assessment of GJ function. These methodological problems have been discussed in details previously (Garcia-Dorado et al., 2004; Végh and Papp, 2011). Nevertheless, despite these difficulties the combination of the available methods and techniques allow us to estimate the function of GJs and their role in arrhythmogenesis under various physiological and pathophysiological conditions.

## THE ROLE OF GAP JUNCTIONS IN ARRHYTHMOGENESIS AND IN THE ANTIARRHYTHMIC EFFECT OF PRECONDITIONING

There were two studies (Smith et al., 1995; Cinca et al., 1997), both performed in anesthetized pigs, which provided the first *in vivo* evidence that GJs play an important role in the generation of the ischemia-induced ventricular arrhythmias. The first study pointed out a relationship between changes in tissue impedance and the occurrence of arrhythmias, showing that the appearance of phase Ib arrhythmias during a 60-min coronary artery occlusion was preceded by a steep increase in tissue resistivity around the 15 min of ischemia (Smith et al., 1995). The second study (Cinca et al., 1997) reported that ischemic preconditioning delays uncoupling of GJs and shifts the onset of the Ib phase arrhythmias to a later period of the ischemia. Our own studies in dogs (Papp et al., 2007) showed somewhat similar results, but the rise in tissue resistivity prior to the occurrence of the phase Ib arrhythmias was not as marked as either in pigs (Smith et al., 1995) or isolated heart preparation (Kléber et al., 1987). Furthermore, preconditioning in dogs not only delayed but significantly decreased the tissue impedance changes (Papp et al., 2007) and, as that we have pointed out previously (Végh et al., 1992a), preconditioning resulted in an absolute reduction in the number and severity of arrhythmias without shifting them to a later period of the occlusion. Preconditioning also preserved GJ permeability and phosphorylation of Cx43 determined both at 25 and 60 min of ischemia, suggesting that preconditioning in this species not only delays but indeed reduces the closure of GJs (Papp et al., 2007). There might be many explanations of these dissimilarities, among which the difference in the preexisting collateral system between dogs and pigs seems to play a major role. This has been thoroughly discussed previously (Végh and Papp, 2011).

Although the mechanisms by which preconditioning influences GJ coupling has not yet been elucidated, it seems reasonable to hypothesize that mediators and signaling pathways, which are thought to play role in this form of cardioprotection, may target and modify GJs, perhaps at the level of connexins. This hypothesis is supported by the fact that GJ channels exist and can switch between various conductance states, which depend on the phosphorylation status of connexins (Kwak et al., 1995; Kwak and Jongsma, 1996). The phosphorylation of the C-terminal of connexins, which determines whether GJs are in open or closed state, involves kinases or kinase-mediated signaling pathways which are activated in response to a preconditioning stimulus. Thus, several kinases, such as protein kinase A (PKA), the various isoforms of PKC, PKG, as well as mitogen-activated protein (MAP) and tyrosine kinases (TK), etc., which have been identified as parts of the preconditioning-induced signaling cascade (Downey et al., 2008), were also shown to target connexins (Dhein, 2004; Salameh and Dhein, 2005). For example, the preconditioning-induced reduction in myocardial damage was associated with a PKC-activated enhanced Cx43 phosphorylation in the rabbit isolated hearts (Miura et al., 2004).

Since the different kinases and kinase isoforms may phosphorylate connexins differently, the resulting responses regarding the regulation of GJ coupling would also be different. Indeed, there are many, sometimes conflicting results reported in both normal and diseased hearts as concerns the activation of a certain kinase pathway and changes in GJ function (Salameh and Dhein, 2005; Dhein et al., 2011). These differences seem to largely depend on the preparations, models and species used, as well as on the experimental conditions applied. Since the regulatory role of the various kinase and signaling pathways on GJs have been excellently discussed previously (e.g., Dhein, 1998; Salameh and Dhein, 2005), it is not purposed to discuss these further. *Nota bene* the exploration of mechanisms which affect GJ function led to the idea that the generation of arrhythmias might be influenced through the modulation of GJs (Dhein et al., 2010).

## PHARMACOLOGICAL MODIFICATION OF GAP JUNCTIONAL COUPLING AND ARRHYTHMIAS

During the past two decades, a number of drugs have been described and developed which facilitate or inhibit the coupling of GJs (reviewed by Dhein, 2004; Dhein et al., 2010). These were used, in part, as tools for obtaining information on the physiological and pathophysiological roles of GJs, in part, as drugs purposing to develop novel antiarrhythmic therapy (Dhein and Tudyka, 1995; Dhein, 2004; Salameh and Dhein, 2005). However, the pharmacological modification of GJ coupling raises also many questions, in particular, when the acute ischemia-induced ventricular arrhythmias are considered. It is still not clear whether opening or closing of GJs during ischemia would be advantageous for arrhythmia suppression. As we, and others (De Groot et al., 2001; DeGroot and Coronel, 2004; Végh and Papp, 2011) have suggested both maneuvers can result in protection. There is no doubt that keeping GJs open during ischemia and thereby maintaining conduction velocity (De Groot and Coronel, 2004) would result in an antiarrhythmic effect. This has been proved by several *in vitro* and *in vivo* studies using synthetic antiarrhythmic peptides, such as AAP10 and rotigaptide (Dhein et al., 1994; Müller et al., 1997; Grover and Dhein, 2001; Xing et al., 2003,^ 2005^; Végh and Papp, 2011). However, more controversial results were obtained with the use of uncouplers, indicating the complexity of the regulation of GJs in both normal and diseased hearts (Garcia-Dorado et al., 1997; Salameh and Dhein, 2005). These differences may be related to the uncoupler used, the model and endpoint examined, as well as the time of administration of the uncoupler to close GJs (Végh and Papp, 2011).

We have experimental evidence that in dogs both the GJ opener rotigaptide and the uncoupler carbenoxolone given prior to and during coronary artery occlusion protected against the ischemia-induced severe ventricular arrhythmias (Végh and Papp, 2011). The fact that the uncoupler carbenoxolone induced an antiarrhythmic effect was indeed surprising, since one would have expected that closing of GJs during ischemia result in enhanced gap junctional uncoupling and arrhythmias. The results of tissue resistivity measurements showed that immediately after the onset of the coronary artery occlusion the decline in phase angle (a measure of increased membrane capacitance due to closure of GJs; Padilla et al., 2003) was more marked in the carbenoxolone treated dogs than in the controls (Papp et al., 2008; Végh and Papp, 2011). Although these early impedance changes are thought not to be attributed to closure of GJs (Kléber et al., 1987), it cannot rule out the possibility that there might be cells within the ischemic area which are severely injured and uncoupled even soon after the onset of the coronary artery occlusion (Wolk et al., 1999; Daleau et al., 2001; Vetterlein et al., 2006). Furthermore, in dogs infused with carbenoxolone the steep increase in resistivity and decline in phase angle that occur usually around the 15 min of the occlusion were also absent. In these dogs the two characteristic arrhythmia phases disappeared, and although ectopic activity could be observed over the entire occlusion period, the total number of ectopic beats was significantly less than in the controls (Végh and Papp, 2011). We proposed that this finding could perhaps be associated with the phenomenon termed “paradoxical restoration of conduction” (Rohr et al., 1997). This suggests that in the border zone, the viable cells are electrically depressed through electrotonic interactions from their neighboring ischemic cells resulting in slowing of conduction (De Groot and Coronel, 2004). However, with the facilitation of uncoupling, such as may occur during ischemia in the presence of an uncoupler, this electrotonic interaction decreases, resulting in an improvement in conduction and, subsequently, a reduction in arrhythmia severity (De Groot and Coronel, 2004). Whatever the precise mechanism is, it seems that carbenoxolone given prior to and during ischemia attenuates impedance changes during the “critical” phase of ischemia and reduces phase Ib arrhythmias, and this effect is similar to that seen with the GJ opener rotigaptide and with preconditioning (Papp et al., 2008; Végh and Papp, 2011).

Interestingly, carbenoxolone almost completely abolished the antiarrhythmic effect of ischemic preconditioning. When it was given prior to and during the preconditioning procedure (two 5-min occlusion and reperfusion insults) both the impedance changes and the ectopic activity were markedly increased during the short ischemic periods compared to the preconditioned dogs without carbenoxolone administration (Papp et al., 2007). In these carbenoxolone treated preconditioned dogs the tissue impedance changes during the prolonged occlusion were as marked as in the non-preconditioned controls, and the severity of arrhythmias, particularly during phase Ib, was also substantially increased. Furthermore, preservation of the phosphorylated form of Cx43 afforded by preconditioning was abolished with the administration of carbenoxolone. Our conclusion was that closing of GJs prior to preconditioning perhaps inhibits the transfer of endogenous substances that are released by the short preconditioning ischemia and reperfusion insults thus inhibiting the activation of signaling pathways leading to cardioprotection (Papp et al., 2007).

As has been mentioned above, many endogenous substances are thought to regulate GJs function by activating various protein kinases (Dhein, 1998; Salameh and Dhein, 2005). Our previous research focused on the exploration of mechanisms involved in the antiarrhythmic effect of ischemic preconditioning, provided substantial evidence that NO is one of the key mediators which plays essential trigger and mediator role in the preconditioning-induced cardioprotection (Végh et al., 1992c). Thus it seemed reasonable to hypothesize that the antiarrhythmic effect of preconditioning and of NO donors (György et al., 2000) may, in part, be accomplished through the modulation of GJ channels.

## EVIDENCE FOR THE ROLE OF NITRIC OXIDE IN THE REGULATION OF CARDIAC GAP JUNCTIONS

The evidence that NO may modulate GJ function comes mainly from studies in non-cardiac tissues (Roh et al., 2002; Patel et al., 2006), especially from those which are dealing with vessel physiology where NO is one of the most important physiological mediators (Kameritsch et al., 2003; Rodenwaldt et al., 2007). These studies showed that NO is able to modify GJ permeability (Bolanos and Medina, 1996; Kameritsch et al., 2003) and the expression of connexin isoforms (Roh et al., 2002; Hoffmann et al., 2003; Yao et al., 2005). This latter would be especially important under chronic conditions where the regulatory role of NO on the expression of connexins has to be considered in terms of the development of chronic heart diseases (Poelzing and Rosembaum, 2004; Akar et al., 2007; Kontogeorgis et al., 2008; Kim et al., 2010; Radosinska et al., 2011). Changes in Cx43 expression play also an important role in the delayed phase of cardioprotection induced by rapid cardiac pacing 24 h prior to ischemia in dogs (Gönczi et al., 2012). In case of the acute and shorter periods of ischemic challenge (such as a 30- to 60-min ischemia) and its arrhythmia consequences, the alterations of GJ conductance, resulting from changes in connexin phosphorylation, seem to be the more likely mechanism through which NO may modify GJ function. However, the signaling pathways, which regulate the level and phosphorylation status of Cx43 and thus modulate the GJ channel properties, are even less well understood in the myocardium than in the other non-cardiac tissues. For example, it has been proposed that stimulation of both α_1_ and β adrenoceptors, although through the activation of different pathways and protein kinases (PKC and PKA, respectively), leads to connexin phosphorylation and to the opening of GJs (Saez et al., 1997; Weng et al., 2002). In contrast, the activation of the guanylyl cyclase-cGMP pathway and the subsequent stimulation of PKG would result in closing of these channels (Dhein, 1998). A more recent study, however, showed that in H9c2 cells, isolated from the rat myocardium, the hypoxia-induced loss in total Cx43 protein content was restored by acetylcholine and also by the administration of the NO donor *S*-nitroso-*N*-acetylpenicillamine (SNAP; Zhang et al., 2006). Since the protective effect of acetylcholine was inhibited by L-NAME, it was suggested that acetylcholine prevents the hypoxia-induced decrease of Cx43 and improves GJ coupling via a NO-mediated pathway.

In our own studies, using sodium nitroprusside (SNP) as an NO donor and administered in intracoronary infusion 20 min prior to and throughout a 60-min occlusion period of the left anterior descending (LAD) coronary artery in anesthetized dogs, we have found that SNP almost completely abolished the severe ventricular ectopic activity and attenuated the increase in tissue resistivity but it did not substantially influence the decrease in phase angle that resulted from occlusion (Gönczi et al., 2009). In the presence of SNP infusion, there was indeed a more marked reduction in phase angle during the first 10-min period of occlusion; and this effect was very similar to that seen with the administration of carbenoxolone (Papp et al., 2008; Végh and Papp, 2011). Furthermore, SNP, like carbenoxolone, abrogated the steep decline in phase angle that occurred in the controls just prior to the appearance of the phase Ib arrhythmias; i.e., the impedance changes remained virtually constant during this critical period of ischemia (i.e., between 15 and 20 min). Despite similarities of impedance changes of SNP and carbenoxolone, these *in vivo* impedance measurements do not provide an answer to the question, as to whether NO, derived from SNP, opens or closes GJs, and whether opening or closing of GJs leads to the antiarrhythmic effect of SNP. However, the fact, that in the presence of SNP the rapid impedance changes that precede the occurrence of phase Ib arrhythmias were markedly attenuated (and in parallel the ectopic activity was virtually disappeared), suggests a preserved GJ function during ischemia and confirms that of our previous supposition that the rate of uncoupling prior to phase Ib is of particular importance in the generation of arrhythmias (Papp et al., 2007; Végh and Papp, 2011). A further evidence that NO may preserve GJ function derived from the *in vitro* measurements. These showed that compared to the controls, SNP maintained GJ permeability and Cx43 phosphorylation even after 60 min of ischemia. In the presence of SNP, the membrane fraction of Cx43 remained largely in phosphorylated form and the metabolic coupling of the adjacent cells was significantly improved. Thus it seems from these results that NO, derived from NO donors, protects the heart against the ischemia-induced early ventricular arrhythmias, and that this effect, at least in part, can be attributed to the effect of NO, or of the NO-stimulated pathways on GJs, as their function is largely preserved in the presence of SNP (Gönczi et al., 2009).

More recent experimental data resulting from the administration of sodium nitrite support this hypothesis. Under experimental conditions sodium nitrite is used as an exogenous nitrite source to prove the importance and the potential therapeutic benefit of nitrite anion. Inorganic nitrites and nitrates, which are natural oxidative metabolites of NO, have been considered for a long time as inert molecules playing not a compelling role in NO physiology. However, over the last decade emerging evidence suggests that inorganic nitrites and nitrates may serve as important reservoirs for NO (reviewed, e.g., Lundberg and Govoni, 2004; Lefer, 2009), since these metabolites, particularly under hypoxic and anoxic conditions, can readily be reduced back to NO (Zweier et al., 1995; Bryan, 2006). This mechanism may provide an increased NO availability under ischemic conditions independently from NO synthase (NOS) activity which is otherwise reduced in the absence of oxygen (Zweier et al., 1995). A number of studies in various experimental animal models have proved that nitrite anion has an important biological function and might represent an effective means to attenuate ischemia and reperfusion injury (e.g., Webb et al., 2004; Duranski et al., 2005; Shiva et al., 2007).

**FIGURE 1:**
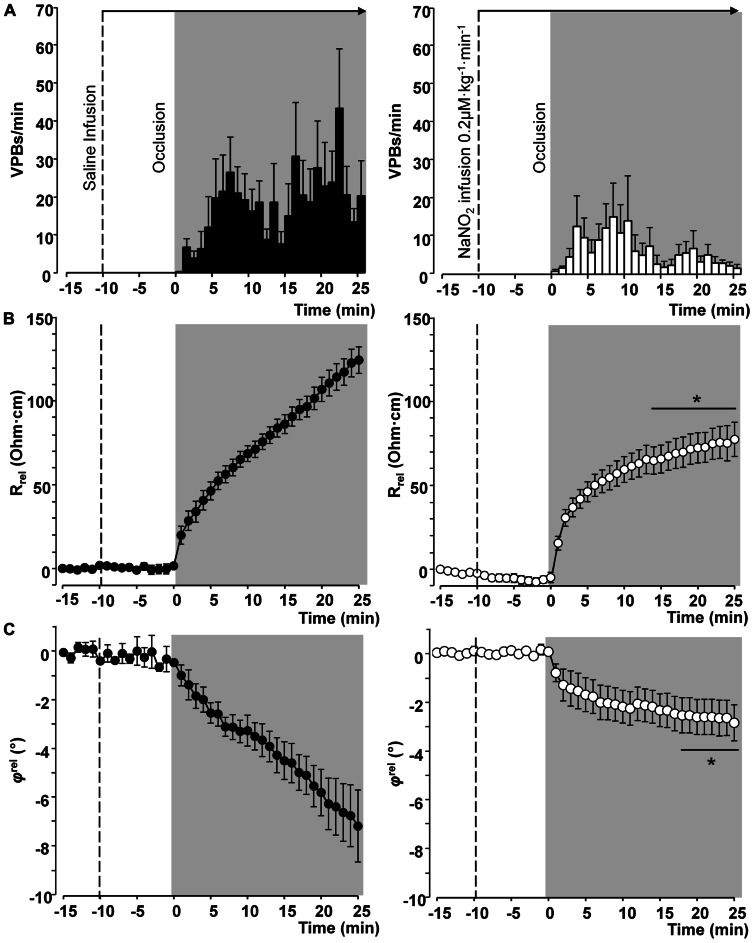
**Distribution of ventricular premature beats (VPBs) and relative changes in tissue impedance (resistivity and phase angle) at one minute intervals during a 25-min coronary artery occlusion in control dogs and in dogs infused with sodium nitrite.** Compared with the controls, the infusion of sodium nitrite markedly reduced the number of VPBs **(A)** and attenuated the rise in tissue resistivity **(B)** and the decline in phase angle **(C)**, particularly during the critical period of ischemia (between 15 and 25 min) when the change of phase angle remained virtually constant. Values are means ± SEM obtained from nine dogs in each group. *P < 0.05 compared with the controls.

**FIGURE 2:**
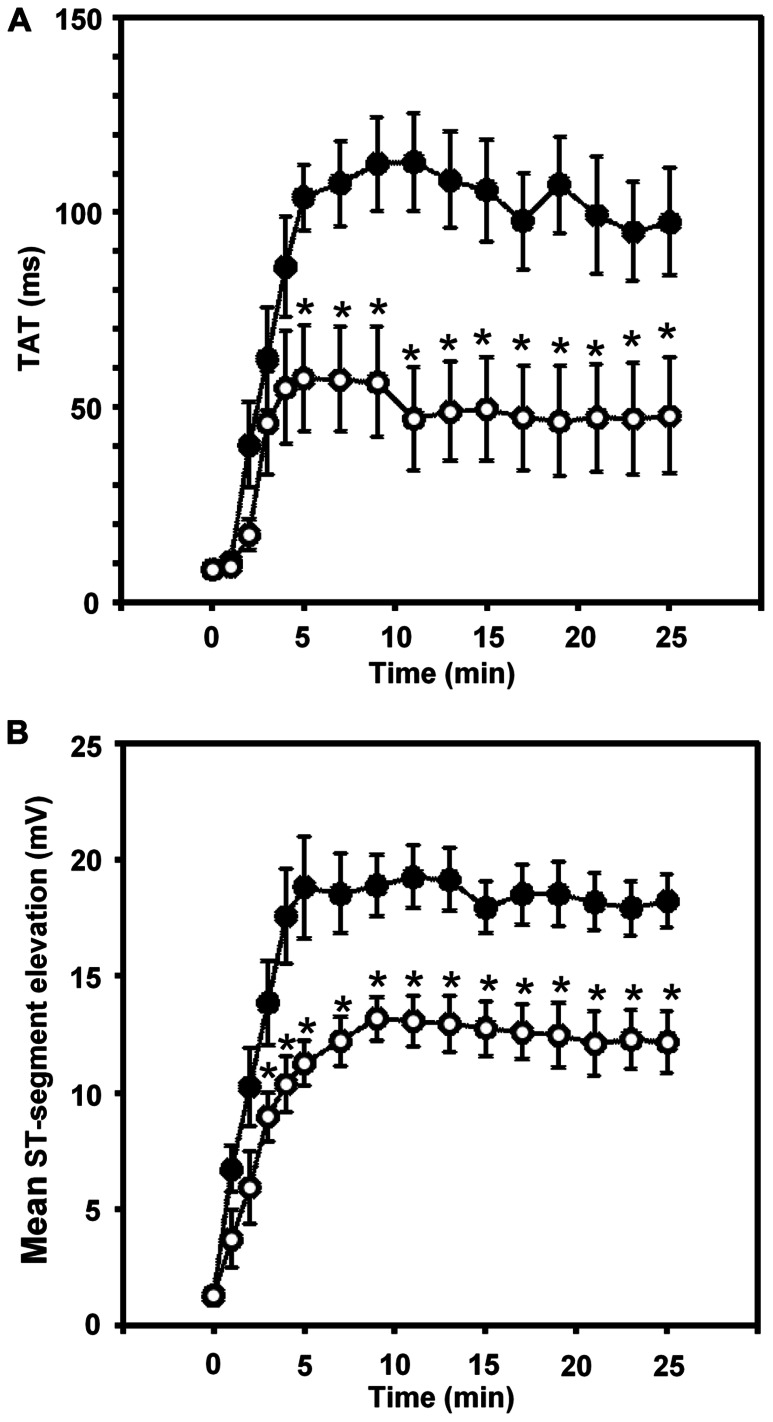
**Changes in the total activation time (TAT) (A) and in the epicardial ST-segment (B) during a 25-min occlusion of the anterior descending branch of the left coronary artery.** In control dogs, both indices of ischemia severity were markedly increased, especially during the initial 5 min of the occlusion. These changes were significantly reduced in the presence of the intravenous infusion of sodium nitrite. Values are means ± SEM. *P < 0.05 compared with the controls.

**FIGURE 3:**
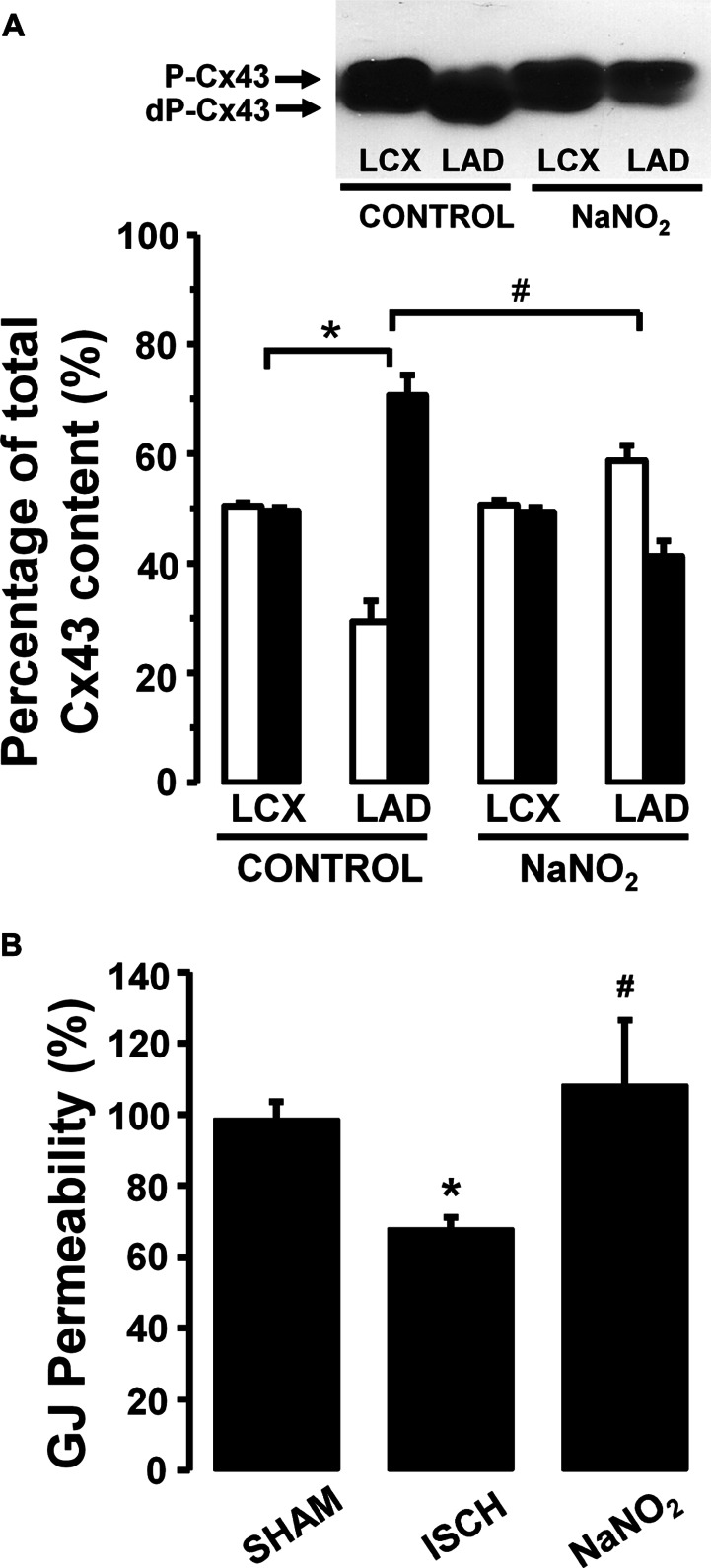
**(A)** A representative Western blot and changes in the phosphorylated (P-Cx43; open columns) and dephosphorylated Cx43 (dP-Cx43; filled columns) isoforms as a percentage of the total sarcolemmal Cx43 content, following a 25-min LAD occlusion The phospho/dephospho ratio within the normal area is around 51/49 ± 1%. This shifted to 29/71 ± 4% in hearts of the control dogs when subjected to a 25-min occlusion. Infusion of sodium nitrite prevented this shift and preserved the phosphorylated form of this protein both within the normal non-ischemic (52/48 ± 1%) and the ischemic myocardial region (59/41 ± 3%). **(B)** Changes in gap junction permeability in sham-control (SHAM) and ischemic control (ISCH) hearts, as well as in hearts infused with sodium nitrite (NaNO_2_). Sodium nitrite prevented the ischemia-induced reduction in gap junction permeability. Values are means ± SEM. ^#^*P* < 0.05 compared with the ischemic control samples. ^*^P < 0.05 compared with non-ischemic samples.

Thus in our anesthetized dog model, we infused sodium nitrite intravenously in a dose of 0.2 μg kg^-^^1^ min^-^^1^, starting the infusion 10 min prior to and maintained throughout the entire 25 min occlusion of the LAD coronary artery, and changes in tissue impedance in parallel with arrhythmia distribution were assessed (Gönczi et al., 2010). We found that in the presence of sodium nitrite infusion the total number of ventricular premature beats during the occlusion was markedly reduced (472 ± 105 vs. 147 ± 77; *P* < 0.05) and the impedance changes were substantially less pronounced than in the controls (Gönczi et al., 2010). This is illustrated in **Figure 1** which clearly shows that in dogs infused with sodium nitrite, the steep increase in resistivity and the decline in phase angle that usually occur around the 14–15 min of ischemia in the control animals were abrogated and the number of ectopic beats during phase Ib was markedly suppressed. In these experiments we also used a mapping electrode, which collects signals from 31 unipolar electrode points of the epicardial surface of the ischemic area in order to evaluate changes in the epicardial ST-segment and in total activation time (TAT) by creating ST and activation maps. The results show that compared with control dogs, in dogs infused with sodium nitrite both the ischemia-induced increases in epicardial ST-segment and TAT were considerable reduced (**Figure 2**). In this study, at the end of the 25 min occlusion period, myocardial tissue samples were taken from the hearts for the assessment of metabolic coupling and Cx43 phosphorylation, as has been described previously (Gönczi et al., 2009). **Figure 3A** shows that the administration of sodium nitrite preserved the phosphorylated form of Cx43 within the ischemic LAD area compared with the control hearts in which the occlusion of the LAD resulted in marked dephosphorylation of Cx43. GJ permeability, determined by double dye loading (Ruiz-Meana et al., 2001; Papp et al., 2007), was also maintained even after the 25 min of ischemia in hearts infused with sodium nitrite (**Figure 3B**).

The results support our previous proposal (Végh and Papp, 2011) that in arrhythmia point of view the modification of GJ function, for example, by preventing the ischemia-induced dephosphorylation of Cx43, would particularly be important during that “critical” phase of ischemia when the rate of uncoupling of GJs rapidly increases, and when other factors, implicated in arrhythmogenesis, are also present. Furthermore, we suggest that NO might be one of the endogenous substances which would regulate GJs not only in vascular tissues (reviewed recently by Looft-Wilson et al., 2012) but also in cardiac myocytes. There is emerging evidence for a cross-talk between NO signaling and connexins in the vasculature which is essential for normal vascular function (Looft-Wilson et al., 2012). Although a strong proof is lacking for such an NO-mediated modulation of GJ proteins in cardiac myocytes, we assume that there might be similar interactions between NO and GJs also within the myocardium, since NO derives either from the “classical” NO donors or inorganic nitrites, or generated during a preconditioning stimulus influenced the electrical and metabolic properties of GJs and resulted in simultaneous alterations in arrhythmia generation. We have proposed previously the most likely scenario for the antiarrhythmic effect afforded by preconditioning is that the preconditioning stimulus triggers the generation and the release of NO from the vascular endothelial cells and also from cardiac myocytes (Parratt and Végh, 1996; Végh and Parratt, 1996). NO by diffusing to cardiac myocytes stimulates soluble guanylyl cyclase and increases cGMP within the myocardium since the inhibition of soluble guanylyl cyclase completely abolished the antiarrhythmic protection (Végh et al., 1992b). cGMP could modify arrhythmogenesis by a number of ways involving the inhibition of calcium entry through L-type calcium channels (Sun et al., 2007), modification of the cGMP/cAMP balance by influencing cGMP-dependent phosphodiesterase and/or the direct depression of cardiac myocytes, resulting in reduced oxygen demand during prolonged ischemia (Parratt and Végh, 1996; Végh and Parratt, 1996). There is evidence that in vascular endothelium both the endogenously produced (Straub et al., 2011) and the exogenously administered (Hoffmann et al., 2003; Rodenwaldt et al., 2007) NO can acutely increase GJ coupling by a cGMP-dependent mechanism. cGMP through the inhibition of the cGMP-dependent phosphodiesterase prevents the degradation of cAMP and stimulates the cAMP–PKA pathway (Francis et al., 2010). This has been shown to enhance the coupling of GJs (Hoffmann et al., 2003). The stimulation of the soluble guanylyl cyclase-cGMP pathway by NO and the subsequent activation of protein kinase G (Patel et al., 2006) might be another signaling mechanism which can lead to connexin phosphorylation and modification of GJ coupling (Lampe and Lau, 2004).

More recent studies suggests that NO can modify GJ function independent from the activation of the NO-induced cGMP–PKG pathway. Such a mechanism is *S*-nitrosylation during which NO reversible binds to the thiol groups of cysteine residue of proteins resulting in *S*-nitrosothiols (SNO). *S*-nitrosylation not only allows the storage and transport of NO (Dejam et al., 2004; Lima et al., 2010) but modulates the activity of several cardiac functions, including cardiac ion channels (Gonzalez et al., 2009), mitochondrial respiration (Sun et al., 2006,^2007^), formation of reactive oxygen species (Sun et al., 2006), or gap junctional connexins (Straub et al., 2011). For example, in the myoendothelial junction, where the vascular endothelial and smooth muscle cells are connected NO has been found to enhance the opening of this special form of GJs through *S*-nitrosylation of Cx43 (Straub et al., 2011). It is reasonable to assume that *S*-nitrosylation of Cx43 would be a possible alternative mechanism by which NO regulates the function of GJs also in cardiac myocytes, especially under conditions of increased NO availability. This may occur, for example, after preconditioning (Kiss et al., 2010), the administration of NO donors (György et al., 2000; Gönczi et al., 2009), including sodium nitrite. There is evidence that *S*-nitrosylation plays an important role in cardioprotection afforded by preconditioning (Sun et al., 2007; Murphy and Steenbergen, 2008). As to whether *S*-nitrosylation of Cx43, indeed, plays a role in the modulation of GJ function by NO and, if so, how much this mechanism accounts for the antiarrhythmic effect is still not known and warrants further examinations.

## SUMMARY

We hypothesized that NO derives from either endogenous (induced by preconditioning) or exogenous sources (administration of NO donors) is able to modulate GJ function, and that this effect of NO, in part, plays a role in the protection against the severe ventricular arrhythmias that results from an acute ischemia and reperfusion insult in anesthetized dogs. To support this hypothesis in the present article we summarized our results obtained from previous and more recent studies which aimed to examine the regulatory role of NO on cardiac GJs in relation to arrhythmogenesis (Gönczi et al., 2009, Gönczi et al., 2010). The results give a strong support for this hypothesis, since in the presence of increased NO availability the function of GJs seems to be well preserved, as have been shown by both the *in vivo* and *in vitro* measurements. These measures, albeit provide only indirect evidence, clearly indicate that a maintained NO availability during a prolonged ischemic insult, resulting from either a preconditioning stimulus or the administration of drugs that liberate NO, inhibits the ischemia-induced tissue impedance changes and dephosphorylation of Cx43, and maintains the metabolic coupling between cells. These effects of NO are especially pronounced during that critical period of ischemia when factors and mechanisms, involved in the generation of the phase Ib arrhythmias are present and fully activated. As a result of the preserved GJ function, the Ib phase of arrhythmias are markedly suppressed. Although the precise mechanisms by which NO attains this GJ modulating effect is still not fully understood, we discussed hypotheses and theories which propose a role for NO in the regulation of GJs. These involve NO-mediated signaling cascades including protein kinases which might have a role in connexin phosphorylation, the classical NO-soluble guanylyl cyclase-cGMP pathway with the subsequent PKG activation and the cGMP-independent mechanism of NO through which NO is able to bind and modify proteins via *S*-nitrosylation. As to whether all these mechanisms are acting together or there is one particular mechanism which preferentially acts under certain circumstances is unknown and requires further investigations.

## Conflict of Interest Statement

The authors declare that the research was conducted in the absence of any commercial or financial relationships that could be construed as a potential conflict of interest.
